# Immunomodulation of wound healing leading to efferocytosis

**DOI:** 10.1002/SMMD.20230036

**Published:** 2024-01-31

**Authors:** Yun Zhao, Minxiong Li, Jiayi Mao, Yinghong Su, Xin Huang, Wenzheng Xia, Xiangfeng Leng, Tao Zan

**Affiliations:** ^1^ Department of Plastic and Reconstructive Surgery Shanghai Ninth People's Hospital Shanghai Jiao Tong University School of Medicine Shanghai China; ^2^ Department of Cosmetic and Plastic Surgery Affiliated Hospital of Qingdao University Qingdao China

**Keywords:** apoptotic cell, efferocytosis, immune cell, phagocytosis, wound healing

## Abstract

Effectively eliminating apoptotic cells is precisely controlled by a variety of signaling molecules and a phagocytic effect known as efferocytosis. Abnormalities in efferocytosis may bring about the development of chronic conditions, including angiocardiopathy, chronic inflammatory diseases and autoimmune diseases. During wound healing, failure of efferocytosis leads to the collection of apoptosis, the release of necrotic material and chronic wounds that are difficult to heal. In addition to the traditional phagocytes‐macrophages, other important cell species including dendritic cells, neutrophils, vascular endothelial cells, fibroblasts and keratinocytes contribute to wounding healing. This review summarizes how efferocytosis‐mediated immunomodulation plays a repair‐promoting role in wound healing, providing new insights for patients suffering from various cutaneous wounds.

## Graphical abstract

Effectively eliminating apoptotic cells is precisely controlled by a variety of signaling molecules and a phagocytic effect known as efferocytosis. Abnormalities in efferocytosis may bring about the development of chronic conditions, including angiocardiopathy, chronic inflammatory diseases and autoimmune diseases. During wound healing, failure of efferocytosis leads to the collection of apoptosis, the release of necrotic material and chronic wounds that are difficult to heal. In addition to the traditional phagocytes‐macrophages, other important cell species including dendritic cells, neutrophils, vascular endothelial cells, fibroblasts and keratinocytes contribute to wounding healing. This review summarizes how efferocytosis‐mediated immunomodulation plays a repair‐promoting role in wound healing, providing new insights for patients suffering various cutaneous wounds.

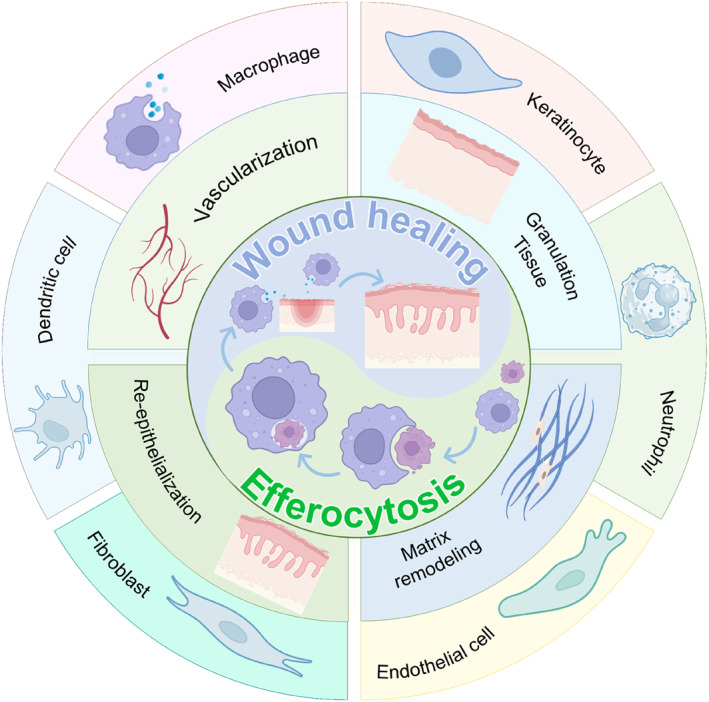

**Key points**

- Key immune cells involved in efferocytosis in wounds are systematically reviewed, as well as the effects of efferocytosis on wound healing.
- This review firstly generalizes the effect of efferocytosis on wound repair and opens ideas for shifting immune control to efferocytosis in wound healing research.
- The review also provides a new strategy for the treatment of chronic difficult‐to‐heal wounds and complex skin defects.

## 1 INTRODUCTION

The skin is the largest organ in the human body, which serves as the body's primary defense against the external context. It can be damaged by a variety of physical and chemical factors, placing a burden on the patient and the social economy.^1^ The repair of skin injuries is a complex and continuous process in which different cell types in the skin act in a synchronized manner.^2^ The speed of repair is determined by the graveness of the injury and the microenvironment to which the cells are exposed during the healing process.

In a healthy organism, cell renewal occurs daily in thousands of cells through programmed death, such as apoptosis and necroptosis.^3^ Dead cells of various types must be efficiently eliminated by specialized or non‐specialized phagocytes.^4^ Specialized phagocytes have macrophages and dendritic cells (DCs), which are endemic in tissues and can continuously absorb dead cells and process them very efficiently. Non‐specialized phagocytes—epithelial cells, fibroblasts, and other stromal cells—can perform similar phagocytosis, but their absorption is slower and less efficient.^5^ Efferocytosis prevents the collection of dead cells and the exudation of intracellular baneful contents, exacerbating inflammatory responses and tissue damage. If efferocytosis is impaired, it will lead to the development of a range of long‐lasting illnesses, but not only atherosclerosis, tumors, diabetic wounds, and other diseases.^6^ Tissue‐resident or recruited phagocytes provide a favorable environment for new tissue growth by effectively removing dead cells from the wound and help to resolve the inflammatory response, promote angiogenesis and matrix remodeling, and restore the skin barrier function. Regardless of the type of cutaneous wound, impaired efferocytosis significantly increases the risk of wound infection and slows healing progress.^7^

**FIGURE 1:**
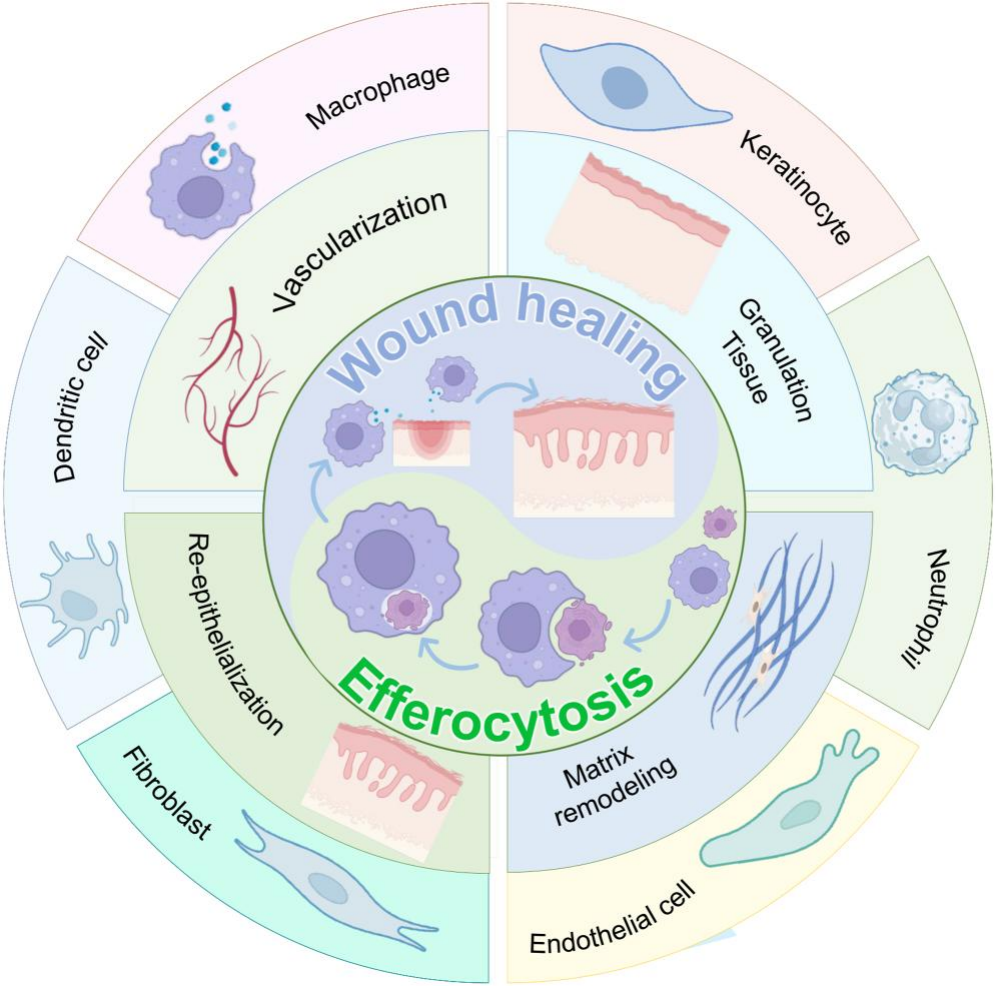
Schematic representation of critical cells participating in efferocytosis to achieve pro‐repair processes for wound healing.

In this paper, we will review the mechanism of efferocytosis during physiological processes and the influence of signaling molecules that may be involved in wound healing and introduce which apoptotic cells (ACs) are generated in the wound and at which points efferocytosis occurs (Figure 1). It is well known that macrophages are the most common phagocytes, in addition to endothelial cells (ECs), keratinocytes, and fibroblasts. In the wound, do they play a similar efferocytosis role? Do they play a similar role in efferocytosis? Can efferocytosis promote wound healing by modulating the inflammatory response, vascularization, and wound matrix remodeling? Next, we outline each of them to provide new ideas for treating different types of wound healing.

## 2 EFFEROCYTOSIS AND WOUND HEALING

**FIGURE 2:**
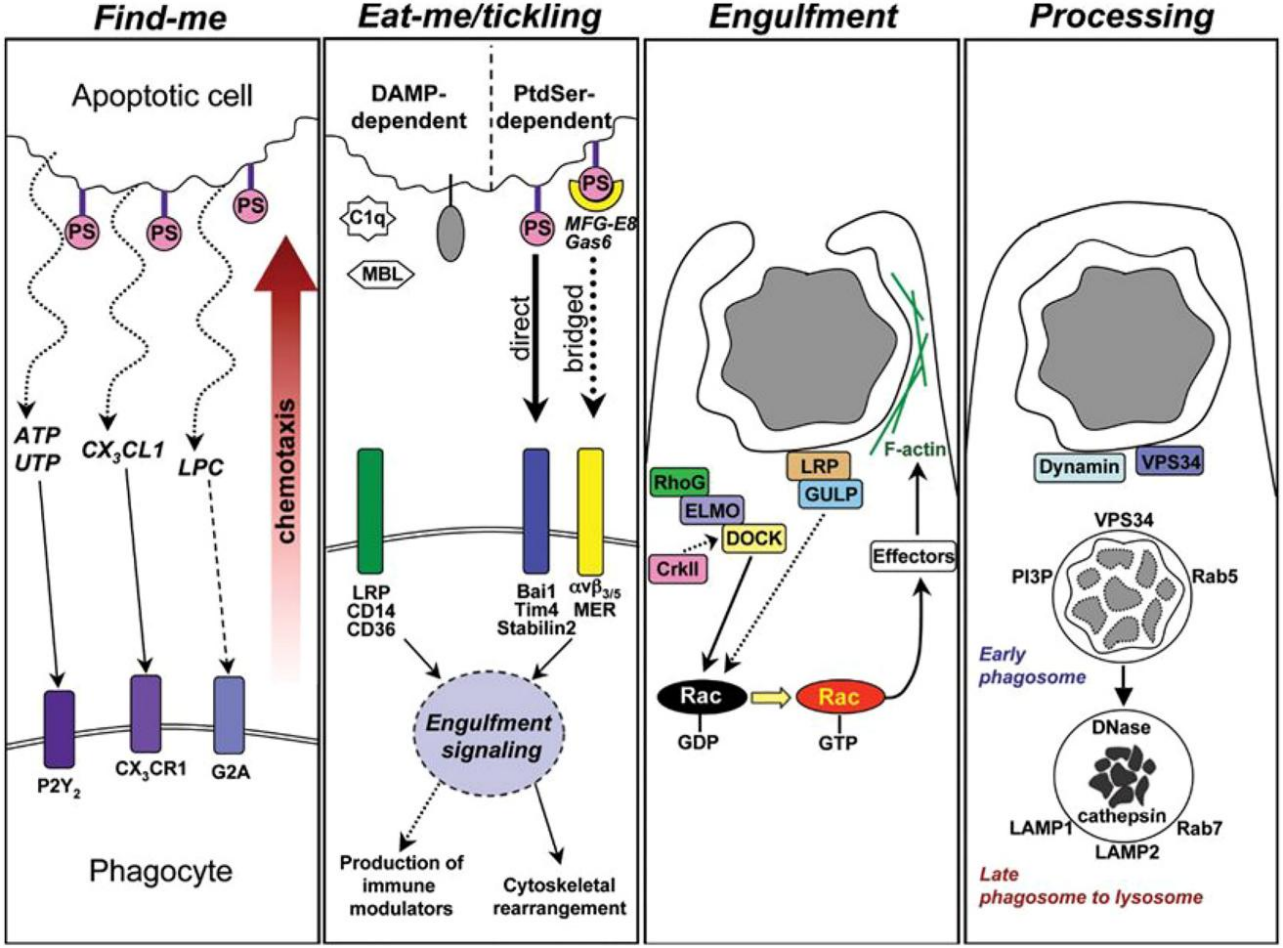
The process of efferocytosis and the key signaling molecules involved. Reproduced with permission.^8^ Copyright 2010, Rockefeller University Press.

The process of efferocytosis is different from the previously recognized phagocytosis through the recognition, binding, encapsulation, and digestion of ACs by phagocytes, which results in the complete elimination of ACs and prevents the secondary necrosis of dying cells and their immune response to autoantigens (Figure 2). Initially, ACs expose specific recognition signals such as lysophosphatidylcholine (LPC), chemokine CX3C ligand 1 (CX3CL1), sphingosine‐1‐phosphate (S1P), nucleotide ATP, and UTP on their surface, resulting in the recruitment of phagocytes to reach the neighborhood.^8^ By binding to G protein‐coupled G2A receptors on macrophages and monocytes, cysteineyl asparagine‐3 activates calcium‐independent phospholipase A2, which releases LPCs, causing ACs to migrate and be engulfed.^9^ CX3CL1 is released from microparticles exposed to phosphatidylserine (PtdSer) and binds to CX3CR1 on phagocytes to induce phagocytosis. ACs release nucleotides (ATP, UTP) from the Pannexin‐1 (PANX1) channel on the plasma membrane, which bind to P2X (ATP‐regulated ion channels) and P2Y (G protein‐coupled receptors) to mediate phagocytic chemotaxis Phagocytosis.^10^ S1P production is associated with sphingosine kinase 2, which binds to S1PR1 on phagocytes and initiate efferocytosis.^11^

When phagocytes reach the vicinity of ACs, they need to distinguish between phagocytized and healthy cells.^12^ This discrimination is achieved through the interaction of ligands on ACs (i.e. “eat‐me signals”) interacting with receptors on phagocytes. On the other hand, apoptotic and living cells do not necessarily express the same signals. To date, several eat‐me signals have been reported on ACs, including PtdSer, calreticulin (CRT).^13^ Among them, PtdSer is the most talked about signaling molecule commonly located on the surface of ACs.^14^ However, phospholipids are asymmetrically distributed across the plasma membrane, and in healthy cells, PtdSer is mainly present in the inner pinole. In contrast, in apoptosis, PtdSer is exposed in the outer pinole of the plasma membrane because cysteine aspartate protease irreversibly cleaves the perforating enzyme XK‐related protein 8.^15^ In addition to PtdSer, certain proteins such as CRT are found on the AC's membrane and act as phagocytic signals.^16^ Ligands on ACs can be identified directly by phagocytic receptors or indirectly sensed by bridging molecules. T‐cell immunoglobulin and mucin‐containing structural domain 4, brain‐specific angiogenesis inhibitor 1, and stabilizing protein 2 directly recognize PtdSer on the surface of ACs, activating downstream signaling and triggering cytoskeletal requirements for AC binding Changes.^17^ Whereas tumor‐associated macrophage (TAM) family members, integrins (e.g., αVβ3 and αVβ5) and CD36 are phagocytic receptors that indirectly recognize PtdSer on ACs by bridging molecules, while CD36 binds directly to oxidized PtdSer.^18^ Platelet reactive protein‐1 (TSP‐1), Gas6, protein S^19^ and milk fat globule epidermal growth factor 8 (MFG‐E8) can connect PtdSer with CD36, TAM members and integrins to play a bridging role, respectively.^20^ Instead, “do not eat me” signaling prevents phagocytosis from removing healthy cells, such as non‐specific signals like CD47 and CD31, thus blocking signaling mediated by signaling regulator protein alpha, which negatively regulates phagocytosis, in phagocytes.^21^

As phagocytes anchor with ACs, they need to rearrange the cytoskeleton and extend pseudopods to encapsulate ACs to form “phagocytic cups,” which in turn engulf membrane‐encapsulated ACs to form phagosomes. The relevant signaling pathway mechanisms involved in actin remodeling from recognition to activation vary from receptor to receptor, but normally demand coordinated activation of kinases (e.g., SRK, SYK, and the protein kinase C family) and phosphatase inactivation.^22^ This series of processes focuses on a key factor, Rac1, a small GTPase of the RHO family, and involves two important mechanisms: the first, mediating the actuation of Rac1 through LRP1 and the junction protein GULP96; the other really depends on the nucleotide exchange factor (GEF) guanine protein dock180 and proteins that regulate phagocytosis, phagocytosis and cell motility proteins (ELMO).^23^ Once phagosomes are formed, they are progressively acidified by sequential recruitment of Rab5 and Rab7, which eventually fuse with lysosomes that degrade ACs,^24^ processing and degradation of harmful substances in ACs.^25^ In addition, since efferocytosis involves the phagocytosis of one cell by another, cellular metabolites including carbohydrates, lipids, proteins, and nucleotides in the phagocyte will be greatly increased in the final phase of efferocytosis, increasing the burden on the phagocyte, but the phagocyte is able to counterbalance the additional metabolic load by self‐activation of degradation and efflux pathways, which is critical in the modulation of the subsequent engulfment by the phagocyte, as well as in the immune response.^26^ At this point, the efferocytosis process was successfully completed and the ACs were removed.

The normal healing process after skin damage in adults consists of four major processes: immediate hemostasis, acute inflammation, granulation tissue and vascularization, and extracellular matrix (ECM) remodeling, which involves the participation of a variety of cells and cytokines. The body's first response to injury is to constrict damaged blood vessels, activate platelets, and initiate coagulation and inflammatory protective mechanisms.^27^ Platelets are inserted into a network of fibronectin, hyaluronan, and platelet reactive proteins to form clots, provide cytokines and growth factors for immune cell recruitment, and act as a protective barrier to the injury environment.^28^ Immune cells and factors play an important role in the early stages of wound healing.^29^ During the inflammatory phase, neutrophils and pro‐inflammatory macrophages play an important role in removing localized pathogens and dead cellular debris through efferocytosis. Among them, neutrophils are the first immune cells to be enrolled in the stopping bolus, initiating the first line of defense for the removal of bacteria and pathogens. Damage from infectious agents is countered by phagocytosis and degranulation, production of high levels of reactive oxygen species (ROS), and formation of neutrophil extracellular traps (NETs).^30^ Another important pro‐inflammatory macrophage, rapidly kills pathogens by recognizing them and internalizing them into intracellular phagosomes, mainly through the acidic environment within the phagolysosome and the high ROS content of the phagolysosome. The mission of neutrophils is accomplished within 3–4 days after injury and they need to be efficiently removed through the efferocytosis action of macrophages to prevent nonspecific tissue degeneration and a persistent inflammatory state.^2^ Next, wound healing enters a proliferative phase during which fibroblasts, macrophages, and ECs participate in angiogenesis and matrix remodeling, and their active cooperation promotes epithelial remodeling, angiogenesis and fibroproliferation.^31^ Details of the relevant signaling molecules of efferocytosis affecting wound healing have been summarized in Table 1.

**TABLE 1:** The role of efferocytosis signaling molecules in wound healing.

Process	Role in efferocytosis	Signaling molecules	Other functions in wound healing
Identify	Find me signal	C‐X3‐C motif ligand 1 (CX3CL1) Lysophosphatidylcholine (LPC) Sphingosine‐1‐phosphate (S1P) Nucleotides (ATP, AMP, UTP)	Regulates angiogenesis^32^ Promotes proliferation and migration of fibroblasts^33^ Promotes collagen deposition^34^ Accelerates wound closure^35^ Regulates angiogenesis^36^ Immune cell phenotype that promotes regeneration^37^ Promotes cell proliferation^38^ Promotes collagen deposition^39^ Increased proliferation of keratinocytes and fibroblasts^40^ Regulates angiogenesis^41^
Bind	Eat me signal	Phosphatidylserine (PtdSer) Calreticulin (CRT) Thrombospondin 1 (TSP1) Milk fat globule‐EGF‐factor 8 (MFG‐E8) Gas6 Protein S	Participate in hemostasis^42^ Promotes the resolution of inflammation^43^ Ca^2+^ homeostasis in vivo^44^ Induces migration of pro‐repair cells^45^ Promotes re‐epithelialization^46^ Regulates fibroblast secretion and collagen deposition^47^ Regulates cell migration and matrix remodeling^48^ Regulates blood clotting and promotes angiogenesis^49^ Promote the migration and proliferation of fibroblasts^50^ Regulates angiogenesis^51^ None
	Bridge molecules		

## 3 CRITICAL CELLS INVOLVED IN EFFEROCYTOSIS FOR WOUND HEALING

As the body's largest immune organ, the skin's immune system has an influence in regulating wound repair. The immune response of the skin is separated into intrinsic immunity and adaptive immunity. Intrinsic immunity (innate immunity) is a natural defense function that humans have. Adaptive immunity is a targeted, evolutionarily advanced immune function that occurs after exposure to antigenic substances.^52^ Depending on the multilayered structure of the skin, immune cells have different distributions. Common immune cells in the epidermis are keratinocytes and Langerhans cells (LCs)^53^; in the dermis, DCs, macrophages, and T cells predominate.^54^

An important step in wound repair is the elimination of cell death induced by the inflammatory environment. This process involves immunomodulation and phagocytosis by specialized phagocytic cells (e.g., internal immune cells, keratinocytes, monocyte macrophages, and DCs), as well as the involvement of T cells mediated by adaptive immune fluids.^55^

### 3.1 Macrophage

Early in the healing phase, it promotes an inflammatory response and removes pathogens, wound debris, and cell death. Later in the healing stage, it inhibits inflammation and releases factors that control the proliferation, and migration of keratinocytes, fibroblasts, and ECs, leading to neovascularization.^56^ Macrophages are known to be the main phagocytic cells of this specialty. The phagocytosis of macrophages is influenced by specific receptor‐ligand interactions, the density of ligands, membrane fluidity and other factors.^57^ The chemokine receptor CX3CR1 is mainly shown by F4/80 macrophages, and CX3CL1‐CX3CR1 can regulate macrophage polarization; Yinhua Ni indicated that CX3CR1 deficiency can lead to an increase in inflammatory monocyte/macrophage infiltration and macrophage polarization toward the M1‐type, exacerbating the progression of non‐alcoholic steatohepatitis.^58^ Valerie et al. noted CX3CR1 deficiency with an increase in lung monocytes and macrophages and a change in macrophage polarization from pro‐repairing to pro‐inflammatory phenotypes.^59^ In conclusion, the above studies suggest that CX3CL1‐CX3CR1 can induce macrophage transformation to a pro‐inflammatory phenotype and exert a pro‐inflammatory phenotype. Liu et al. suggested that LPC induces macrophage migration, increases the creation of pro‐inflammatory cytokines, induces oxidative stress, promotes apoptosis and contributes to the development of disease.^60^ Hou et al. have found that S1P can promote NLRP3 inflammatory vesicle initiation and actuation in a dose‐dependent manner centered on macrophages to promote liver fibrosis.^61^ Astrid et al. reviewed the possible role of macrophages during tumor progression and showed that macrophages can produce pro‐vascular growth factors, promote tissue repair, and regulate tissue remodeling.^62^

### 3.2 Dendritic cell

**FIGURE 3:**
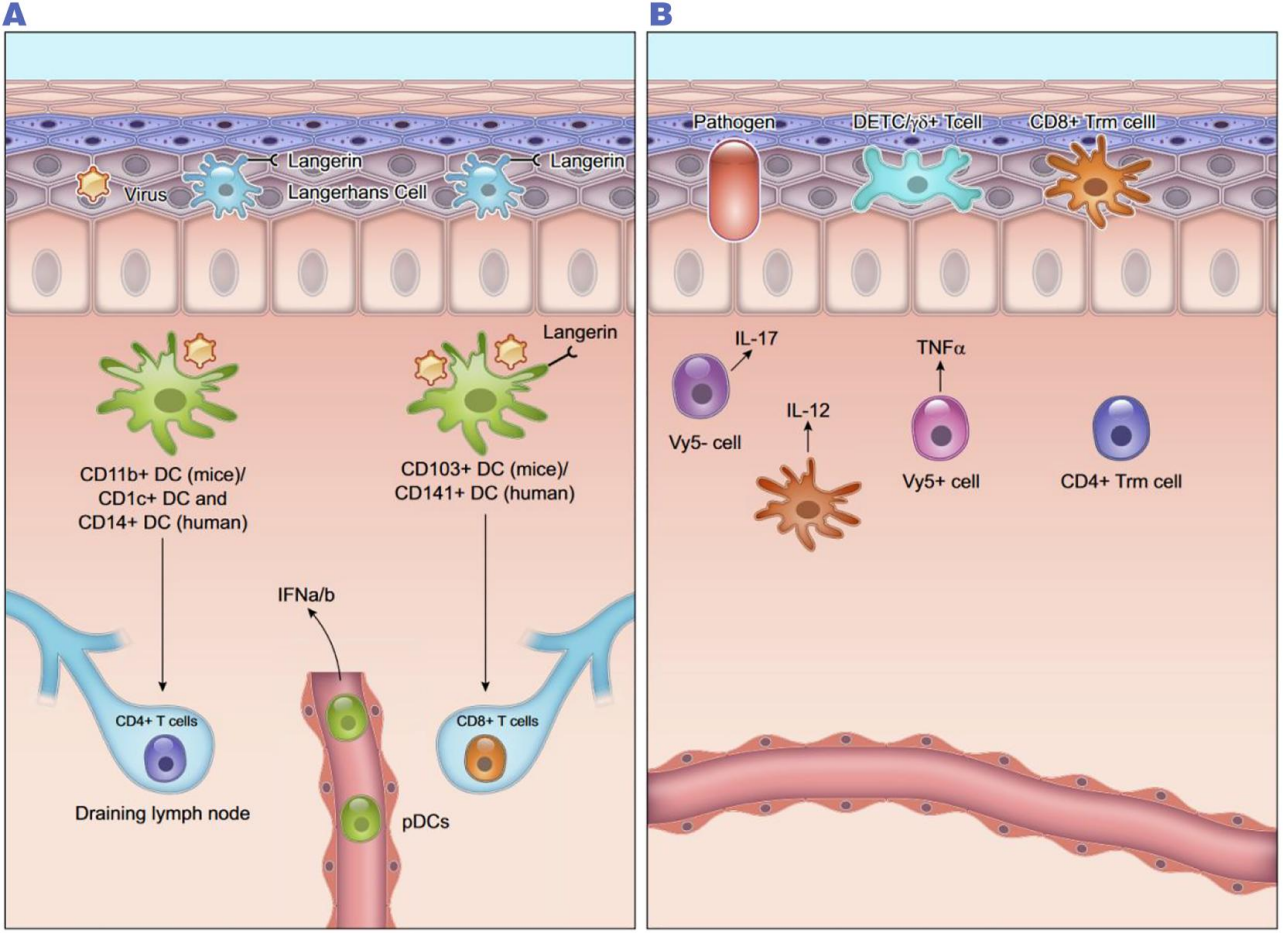
The role of DCs in cutaneous wounds. (A) DCs types in dermis. CD103 DCs predominantly activate CD8 T cells and CD11b DCs predominantly activate CD4 T cells. Injured skin also contains plasma cell‐like DCs (pDCs), which are activated to release interferon (IFN). (B) T cells of the epidermis are also known as dendritic epithelial cells (DETCs). These cells investigate epidermal infections and produce growth factors. Reproduced with permission.^2^ Copyright 2019, The American Physiological Society. DCs, dendritic cells.

DCs are specialized antigen‐presenting cells and are divided into mature and immature DCs depending on the function they perform. DCs are mainly regulated by DAMP (danger‐associated molecular patterns), complement proteins, lipids, and chemokines to enable them to correctly localize and chemotactically.^63^ The DCs of the skin are divided into two distinct subpopulations: the epidermis is dominated by LCs, which migrate and differentiate early in the life cycle of DCs and act as sentinels of the immune system to obtain antigens from the stratum corneum; and the dermis is dominated by the traditional DCs (cDCs), which are divided into cDC1 (which have a high turnover rate) and cDC2 (which are most prevalent in the dermis), as well as a few Mo‐DCs.^64^ Similar to the role of macrophages, DCs have the ability to regulate the inflammatory response by releasing cytokines and chemokines as well as phagocytosis to kill pathogens, helping to maintain tissue homeostasis.^65^ The impaired function of DCs, reduced phagocytosis of pathogens, diminished antigen presentation (APC), and failure to effectively relieve tissue inflammation greatly affect wound healing. Therefore, it is crucial to understand the mechanism of action of DCs to effectively phagocytose ACs/pathogens and how their efferocytosis affects wound healing (Figure 3).

DCs and macrophages are well known as specialized phagocytes with strong devouring ability in peripheral tissues and lymphoid organs, but DCs exert phagocytosis by different mechanisms; macrophages can instantly clear pathogens in situ, whereas the clearance of DCs depends on their growth status. Phagocytically competent DCs, referred to as immature DCs, express lectins, scavenger receptors, and pathogen receptors, and exert immediate pathogen clearance.^66^ Phagocytosis of immature DCs relies on the delivery of antigens from the endocytosed vesicular network from the phagosome to the lysosome for degradation, which contains a variety of esterases, reductases, and endopeptidases that are regulated by environmental pH.^67^ Recent studies have found that the efferocytosis action of DCs has an influence on diabetic wound healing, which presents a new perspective on the contribution of efferocytosis action to wound healing. Maschalidi et al. stated in a paper published in Nature that high expression of the SLC7A11 gene has been found in diabetic cutaneous trauma DCs, and that SLC7A11 can act as a DC mediating the mediation of efferocytosis effects, DC efferocytosis effects were enhanced upon inhibition of SL7A11 under high apoptotic load, and tissue repair was stimulated by promoting AC clearance and secretion of transforming growth factor β superfamily member GDF15.^68^ Juban et al. showed that stimulating the efferocytosis action of DCs accelerates wound healing by accelerating the clearance of traumatic skin debris.^69^ The presence of DCs in peripheral tissues and their ability to secrete them efficiently offer the possibility of absorbing foreign and pure antigens during homeostasis or infection in vivo.^70^

### 3.3 Neutrophil

After wound injury, tissues release DAMPs, chemokines such as CXCL8, and growth factors such as platelet‐derived growth factor and vascular endothelial growth factor (VEGF) ‐A in order to recruit neutrophils, macrophages, and monocytes, to arrive at the wound site and initiate an inflammatory response.^71^ Neutrophils are the first cells to recruit to the injury site and respond to infectious agent damage by phagocytosis and degranulation, high ROS production and extracellular trap (NET) formation.^30^ It has been shown that a decrease in the number of neutrophils recruited by the wound will greatly affect wound healing.^33^ NET is encapsulated by histones, cytoplasmic proteins and proteases that trap and eliminate exogenous pathogens.^72^ The main process is that neutrophils first ingest the pathogen and then fuse with intracellular particles containing hydrolase and NADPH oxidase subunits to form mature phagosomes, which initiate the killing mechanism.^30^ The maturation of phagosomes is not always smooth, and when phagocytotic particles fuse with phagosomes prematurely, or when large number of immune complexes are deposited on the surface of the phagosome, phagocytosis fails and triggers inflammatory diseases leading to tissue damage.^73^ With the initial anti‐inflammatory effect of neutrophils, it will be faced with the apoptosis of these neutrophils, which initiates their phagocytosis by macrophages through their release of PtdSer, which not only increases the phagocytic activity of macrophages, but also polarizes them to a pro‐repair phenotype, provides a good basis for subsequent tissue repair.^74^

### 3.4 Endothelial cell

It is important for vascular ECs to regulate vascular tone, act as a mechanical barrier between the vessel wall and blood flow, and induce angiogenesis.^39^ ECs have been shown to have the ability to not only participate in APC, but also to induce apoptosis, they can phagocytose macromolecular waste products generated by the circulation, and they participate in efferocytosis.^75^ Several studies have demonstrated that ECs also have some ability to phagocytose ACs, and ECs from the liver, spleen, and other sites can complement phagocytosis by macrophages.^76^ There is endothelium dysfunction, such as a reduction in diastolic factor release, a surplus of contractile factors, and a proinflammatory response, apoptosis and cellular motility imbalance can cause vascular injury and worsens peripheral arterial disease. Understanding the effect of phagocytosis of ECs and their efferocytosis on wound healing is crucial in the proliferative phase of re‐invasive wound healing.

Under physiological conditions, ACs are mainly cleared by specialized phagocytosis such as macrophage cells; however, in vascular diseases such as vasculitis caused by anti‐neutrophil cytoplasmic antibody or atherosclerosis, when specialized phagocytosis cannot clear ACs or their number is large enough, phagocytosis is delayed, and then the ECs will activate the phagocytosis to help to clear the excess debris.^77^

### 3.5 Epithelial cell

The epithelial tissue plays a crucial role in initiating and controlling the initial attack of microbial pathogens.^78^ Numerous studies have indicated that epithelial cells also have some phagocytosis. Si et al. indicated that the retinal pigment epithelium (RPE) is a highly specialized epithelium, and that phagocytosis of photoreceptor (POS) cells by the RPE ensures continuous renewal of photoreceptor cells, which maintains daily photoreceptor renewal as well as capillaries nutrients and functions.^79^ Kailin et al. used live microscopy to non‐invasively visualize live cell behavior during hair follicle regression in mice and found that basal epithelial cells phagocytose and remove adjacent epithelial cell debris.^80^ Monks et al. found that live mammary epithelial cells can phagocytose ACs in degenerating mouse mammary glands, and it has been shown in vitro cultures that many of the same receptors used by macrophages are also utilized by MECs during phagocytosis of ACs, including CD36, the receptor avb3, and CD91. They produce proinflammatory cytokines similar to macrophages, which are crucial to controlling inflammation.^81^ Satoko et al. indicated that kidney injury molecule (KIM)‐1 enhances phagocytic removal of debris by epithelial cells and contributes to renal tissue repair by studying a model of acute kidney injury. It is clear from these studies and findings that phagocytosis by epithelial cells plays an important role in maintaining the function of a cell and repairing the tissue.

### 3.6 Keratinocyte

The keratinocyte plays a crucial role in wound healing, not only as a structural cell, but also as a key immune cell. Through APC, keratinocytes can directly interact with T cells. Additionally, antimicrobial peptides produced by keratinocytes can kill invading pathogens directly, which contributes to wounding healing.^82^ Francesca et al. in vitro phagocytosis of human keratinocytes using fluorescent latex beads found that the tyrosine kinase FGFR2b/KGFR triggered phagocytosis and melanosome transfer in differentiated keratinocytes.^83^ Human keratinocytes can phagocytose a variety of cytokines, and Monica et al. demonstrated that fibroblast growth factor receptor 2 (FGFR2b) induces this process.^84^

### 3.7 Fibroblast

As a key component of tissue growth and development, fibroblasts are responsible for producing and remodeling ECM in the body's connective tissues.^85^ Upon being stimulated by inflammatory signals, fibroblasts differentiate into myofibroblasts, which exhibit an increase in cell proliferation, migration, and smooth muscle expression, when the stimulation persists excessive cytokine, vasoactive peptide, and growth factor release leads to pathological remodeling and fibrosis.^86^

There have been many reports in the literature defining fibroblasts as non‐specialized phagocytes that were found to phagocytose apoptotic neutrophils as early as 1994. By phagocytosing apoptotic neutrophils, ECs, or other necrotic cells, fibroblasts provide the necessary conditions and environment for the growth of new tissues. According to Banergee et al., the chromatin protein HMGB1 plays a role in the efferocytosis of ACs by fibroblasts. Their findings showed that inhibition of HMGB1 increased apoptotic thymocytes and apoptotic neutrophils uptake via Rac1‐mediated FAK‐Src activation.^87^ Based on Hermetet et al.'s findings, fibroblasts identify and phagocytose late ACs and early ACs, respectively, and that they phagocytose ACs in a pattern that resembles that of early ACs, macrophages are more efficient and faster at removing ACs than fibroblasts.^88^ All these studies affirm the phagocytosis of fibroblasts and their contribution to the clearance of ACs and tissue repair.

### 3.8 T‐cell

How do adaptive immune cells, T cells, function as cytosolic burials? T cells are divided into CD4+ T cells (helper T cells) and CD8+ T cells (cytotoxic T cells). Helper T cells are further divided into five types: Th1, Th2, Th17, regulatory T cells (Treg) and follicular helper T cells. Among them, IFN‐γ and TNF‐α produced by Th1 can activate macrophages, which produce NO to promote the fusion of phagosomes and lysosomes and release various antimicrobial substances.^89^ At the same time, activated macrophages can secrete IL‐12 to promote the differentiation of Th1 cells, expanding the response of Th1 cells and forming a positive feedback effect.^90^ Th1 can also secrete cytokines, such as IL‐3 and granulocyte‐macrophage colony stimulating factor, to induce the differentiation of bone marrow stem cells into macrophages, and produce cytokines, such as TNF‐α, TNF‐β and so on, which can induce macrophage chemotaxis to the site of infection, enhancing the killing effect of macrophages.^91^ Th1 can also play a role in T cells, B cells and neutrophils by inducing B cells to produce antibodies that have a stronger physiological effect, such as IgG2a, which binds to the antigen and enhances phagocytosis of pathogens by macrophages.^92^ While the TNF‐α produced by Th1 can promote the activation of neutrophils and similarly enhance their pathogenic effect.^93^ Helper T cells activate macrophages by secreting cytokines and enhance their chemotaxis, which enhances their phagocytosis and promotes their burial. In atherosclerosis, Treg cells stimulate macrophage production of IL‐10 by secreting interleukin‐13 (IL‐13), and autocrine‐paracrine signaling of IL‐10 induces vav guanine nucleotide exchange factor 1 in macrophages, which activates Rac1 and facilitates macrophage cytokinesis.^94^ In contrast, cytotoxicity exerts a direct killing effect on target cells. Next, it will be described how cells other than adaptive immune cells, T cells, play a role in cytokinesis through immunomodulation.

## 4 THE EFFECT OF EFFEROCYTOSIS IN WOUND HEALING

When the tissue is cleared of necrotic cellular debris and dead cells, a series of subsequent pro‐repair processes are activated, typically represented by vascularization, re‐epithelialization, and matrix remodeling, in which the signaling molecules, pathways, and cells involved play a synchronized role.

### 4.1 Vascularization

In order for wounds to heal effectively, new blood vessels must be formed or neovascularization must occur. Cell proliferation and tissue regeneration are dependent on nutrient delivery and oxygen homeostasis.^95^ Neovascularization occurs in two steps: vascular sprouting and then vascular anastomosis. It is not only anti‐inflammatory and repair‐promoting macrophages that release VEGF, a hormone that promotes vascular sprouting, but they also express two proteins that promote anastomosis among arteries.^96^ Vascular ECs are important cells involved in angiogenesis and act as gatekeepers to protect underlying tissues from blood‐borne toxins and pathogens by phagocytosing fibrin clots and cellular debris in the bloodstream.^97^ In addition, chemokines involved in efferocytosis can also be involved in the formation of blood vessels in wound healing. Among them, CX3CL1 can increase the proliferation and migration of HUVECs and stimulate angiogenesis in vivo through the activation of G protein‐coupled receptor.^98^ S1P maintains endothelial integrity and prevents vascular leakage, and it stimulates angiogenesis.^99^ Aside from promoting EC survival, it acts as a chemoattractant for vascular cells as well as protecting the endothelial barrier.^100^ MFG‐E8 is a pro‐angiogenic factor that translocates and accumulates around damaged blood vessels and enhances wound healing during vascular injury or damage.^101^

### 4.2 Granulation tissue generation

Type III collagen, fibroblasts, and neovascularization make up the granulation tissue, and its formation occurs simultaneously with angiogenesis. Granulation tissue formation is mainly involved by fibroblasts, and some macrophage‐derived molecules, such as TNF‐α, IL‐1, and IL‐6, induce pro‐epithelialization molecules in fibroblasts.^102^ Granulation tissue can simultaneously initiate macrophage polarization toward pro‐restorative polarization, fibroblast proliferation and myofibroblast differentiation, followed by wound contraction, neointimal formation and matrix deposition.^103^ Poor production of granulation tissue can lead to chronic healing or even non‐healing of the wound; excessive deposition of granulation tissue, such as over‐proliferation of fibroblasts and/or persistence of myofibroblasts in the granulation tissue can lead to scar formation,^104^ At this point, phagocytes play a role in preventing excessive deposition of collagen fibers. It has been suggested that the “eat‐me” signal CRT can promote the formation of traumatic granulation tissue.^46^ However, there is no direct evidence on other effects of phagocytosis or efferocytosis on trabecular granulation tissue production, mostly indirectly by regulating the metabolism of fibroblasts or myofibroblasts.

### 4.3 Re‐epithelialization

**FIGURE 4:**
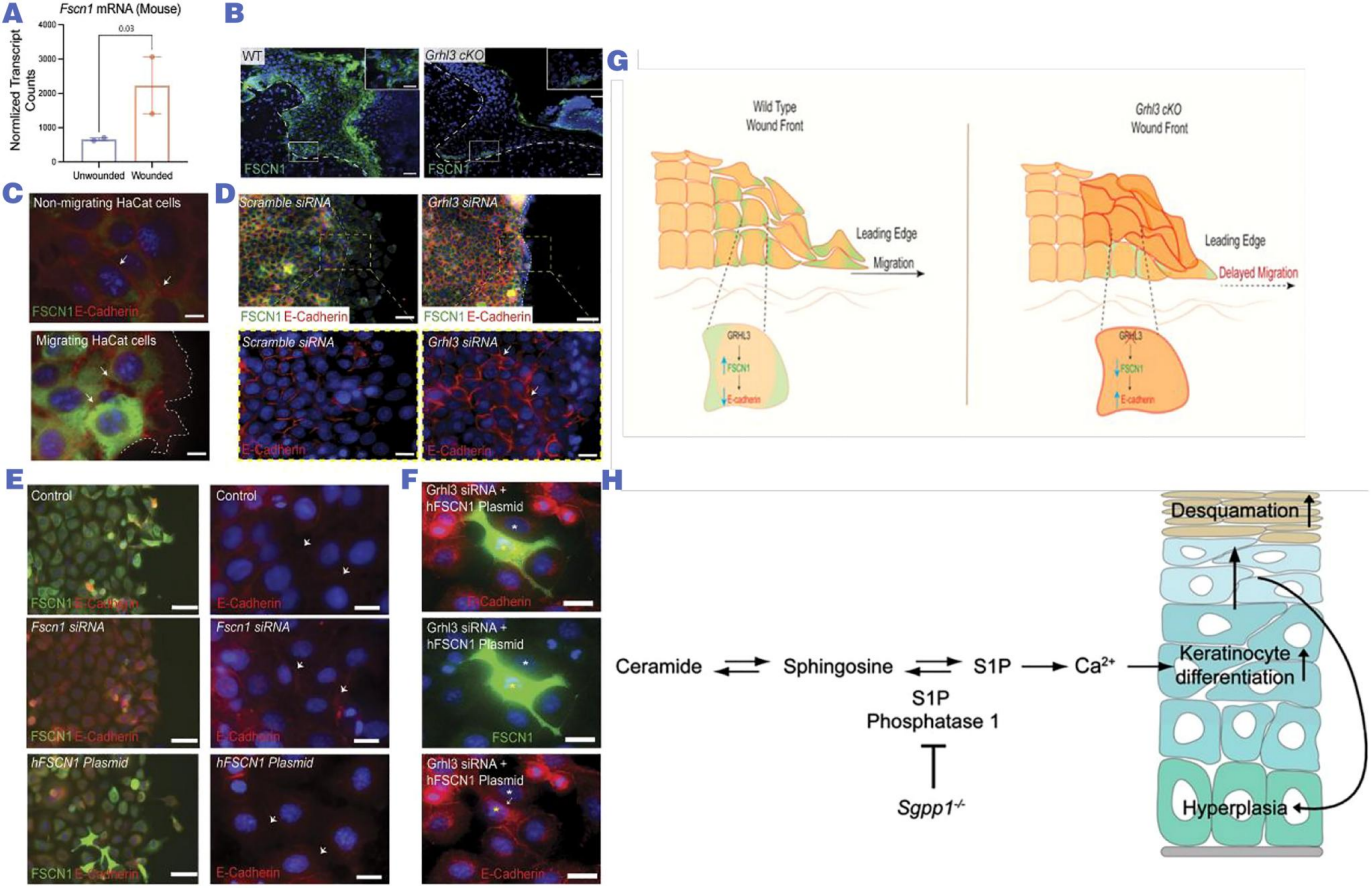
(A–G) GRHL3 upregulates FSCN1 in migrating pre‐wound keratinocytes, and FSCN1 increases keratinocyte migration, which is important for wound re‐epithelialization. Reproduced under terms of the CC‐BY license.^105^ Copyright 2021, The Authors, published by the American Society for Clinical Investigation. (H) In addition, S1P signaling molecules can inhibit keratinocyte proliferation and induce keratinocyte differentiation and migration to promote wound healing. Reproduced with permission.^106^ Copyright 2013, ASBMB. FSCN1, fascin actin‐bundling protein 1; GRHL3, grainyhead like 3; S1P, sphingosine‐1‐phosphate.

As the first line of defense of the organism, the integrity of the epidermis plays a decisive role in the barrier function of the skin. When the skin is superficially injured, keratin‐forming cells accomplish this by proliferating, migrating, and covering the wound; when the damage is deeper, the epidermis begins to regain its integrity after granulation tissue filling. During epithelialization, keratinocytes, which constitute the outermost layer of the skin, migrate to the top of the newly formed granulation tissue, and the GRHL3 (grainyhead like 3)/FSCN1 (fascin actin‐bundling protein 1)/E‐cadherin pathway is activated in the cells of the injured skin (Figure 4), and these keratinocytes meet with keratinocytes from the contralateral edge, these keratinocytes meet migrating keratinocytes from the opposite edge, and the wound closes.^105^ Growth factor‐induced re‐epithelialization occurs when epidermal growth factor (EGF) is generated by platelets, keratinocytes, and adipocytes, respectively, meanwhile Anti‐inflammatory macrophages are activated as well as pro‐repairing macrophages.^107^ As a “find me” signal, S1P inhibits keratinocyte proliferation, induces keratinocyte differentiation and migration, and plays a role in wound healing.^100^ Through its interaction with the P2 purinergic receptor, extracellular ATP induces the release of heparin‐binding EGF‐like growth factor from keratinocytes. It stimulates keratinocyte proliferation and migration by binding to EGF receptors, and plays an essential role in re‐epithelializing cutaneous wounds.^108^ CRT also undergoes a concentration‐dependent migration by inducing keratinocytes, which is important for its effect on re‐epithelialization.^81^

### 4.4 Matrix remodeling

Stromal remodeling is the final stage of wound healing and determines the further progression of the wound toward scarring or non‐healing. During remodeling, neovascularization is suppressed, the ECM is deposited cyclically, and scar tissue is reconstructed out of granulation tissue. The wound granulation tissue consists mainly of collagen III, which is slowly replaced by collagen I during wound remodeling.^2^ With the completion of wound healing, myofibroblasts mediate apoptosis in the granulation tissue, which, if impaired or dysfunctional, will lead to the progression of the wound in the direction of scar formation.^109^ Pro‐repair macrophages have fibrinolytic functions and inhibit excessive matrix deposition by breaking down excess ECM, phagocytosing ECM debris and ACs. It was found that an increase in the efferocytosis‐associated chemokine CX3CR1 promoted the accumulation of myofibroblasts and led to collagen deposition.^34^ S1P inhibits collagen production by dermal fibroblasts, resulting in smaller granulation tissue and thin scars when re‐epithelialization is complete.^110^ CRT has been shown to regulate fibroblast ECM expression, secretion and production, thereby inducing matrix remodeling.^111^

## 5 SUMMARY AND OUTLOOK

This article highlights the relationship between efferocytosis and wound healing. Efferocytosis is mainly mediated by immune cells during the inflammatory phase of wound healing, but it is by no means limited to this phase. The efferocytosis of re‐epithelialization and matrix remodeling can be mediated by phagocytosis of apoptotic or redundant fibroblasts, myofibroblasts, and ECs in wounds, either to provide sufficient space for the neoplastic cells or to prevent their over‐accumulation, which can impair the progression of wound healing. As wound healing involves a variety of cells, we have found that a wide range of cells involved in wound healing can exert phagocytosis, and that signaling molecule associated with efferocytosis can also interact with wound ECs, fibroblasts, and neutrophils and differentially affect their re‐epithelialization, vascularization, matrix remodeling, and other functions. Although the role of efferocytosis has been studied in cardiovascular, oncological, and hepatic contexts in the past decades, little is known about its role in wound healing. In the future, it may be necessary to explore in greater depth the factors through which efferocytosis can influence the progress of wound healing and provide new ideas for the treatment of acute and chronic wounds.

## AUTHOR CONTRIBUTIONS

Tao Zan and Wenzheng Xia conceived the study. Yun Zhao wrote the manuscript. Minxiong Li, Jiayi Mao, Yinghong Su, Xin Huang and Xiangfeng Leng helped revise the manuscript. All authors have read and approved the final manuscript.

## CONFLICT OF INTEREST STATEMENT

All authors declare that there are no competing interests.
